# Rethinking the hepatoprotective potential of vegetarian diets in dysfunction-associated steatotic liver disease/metabolic-associated fatty liver disease: a critical narrative review

**DOI:** 10.3389/fnut.2026.1902349

**Published:** 2026-07-15

**Authors:** Wentong Jiang, Ziyao Yang, Yixin Cui, Nafei Huang, Lisha Dong, Tao Sun, Bing Zhang

**Affiliations:** 1 The Second Clinical Medical College of Zhejiang Chinese Medical University, Hangzhou, Zhejiang, China; 2 Department of Hepatology, The Second Affiliated Hospital of Zhejiang Chinese Medical University, Hangzhou, Zhejiang, China; 3 Department of Traditional Chinese Medicine, Hangzhou First People’s Hospital, Hangzhou, Zhejiang, China

**Keywords:** gut-liver axis, hepatoprotection, metabolic dysfunction-associated steatotic liver disease, metabolic-associated fatty liver disease, plant-based diet quality, precision nutrition, vegetarian dietary patterns

## Abstract

Dietary intervention is central to the management of metabolic dysfunction-associated steatotic liver disease (MASLD), previously known as metabolic-associated fatty liver disease (MAFLD). Although vegetarian dietary patterns are widely considered hepatoprotective, emerging evidence indicates that certain individuals following non-standardized vegetarian diets may fail to obtain the expected hepatic benefits—a phenomenon termed the “vegetarian hepatoprotection paradox.” This critical narrative examines the putative mechanisms underlying this paradox. Excessive intake of high-glycemic carbohydrates and fructose, which is common in some vegetarian diets, may activate hepatic *de novo* lipogenesis. Furthermore, incomplete plant proteins may contribute to impaired very low-density lipoprotein (VLDL)-mediated lipid export, while an imbalanced n-6:n-3 fatty acid ratio may promote hepatic inflammation. These dietary factors may interact with genetic susceptibility, including patatin-like phospholipase domain-containing protein 3 (PNPLA3) I148M and transmembrane 6 superfamily member 2 (TM6SF2) E167K variants, disrupting hepatic lipid homeostasis. Additionally, gut dysbiosis may propagate metabolic disturbance through chronic inflammation. Accordingly, a shift from the categorical vegetarian labeling toward an emphasis on plant-based dietary quality—reflecting the principle that dietary quality matters more than the vegetarian label itself—may be warranted. This review discusses evidence-based dietary patterns, including the Mediterranean diet, the Green Mediterranean diet, and the Dietary Approaches to Stop Hypertension (DASH) diet, together with chrononutrition strategies. A precision nutrition approach integrating genetic, metabolic, and microbiome characteristics may represent an emerging but still investigational framework for individualized MASLD/MAFLD interventions.

## 1 Introduction

Historically described as non-alcoholic fatty liver disease (NAFLD), it was renamed as metabolic dysfunction-associated fatty liver disease (MAFLD) in 2020 (1) and subsequently metabolic dysfunction-associated steatotic liver disease (MASLD) in 2023 (2). This review uses the dual designation “MASLD/MAFLD” throughout the text to maintain consistency with cited sources, which were published during the period when all three terms were in use (1, 2). Current epidemiological data indicate that MASLD/MAFLD affects approximately 1 billion individuals worldwide (3–5). MASLD/MAFLD usually progresses from hepatic steatosis to steatohepatitis, progressive fibrosis, cirrhosis, and then hepatocellular carcinoma (6–8). However, MASLD/MAFLD is recognized as the hepatic manifestation of systemic metabolic disorder, not limited to the liver. Furthermore, cardiovascular disease is currently the leading cause of mortality in MASLD/MAFLD patients, accounting for approximately 40–50% of deaths (9, 10). Additionally, MASLD/MAFLD is correlated with an elevated risk of extrahepatic malignant tumors (11). Chronic inflammation and immune-metabolic imbalance in MASLD/MAFLD increase the risk of several extrahepatic neoplasms and immunotherapy-dependent adverse events (12, 13). Considering these issues, the broad-ranging effects of MASLD/MAFLD have incurred significant economic losses in direct medical expenditure, such as the prolonged use of drugs, complication management, and the cost of treatment involving end-stage liver transplantation. Modeling estimates suggest the annual direct medical expenses related to NAFLD of about 103 billion United States (US) dollars in the US and around 35 billion euros in Europe. Beyond these figures, the related long-term issues, such as morbidity, disability, and reduced lifespan, indirectly impose an even greater amount of cost. Nevertheless, insufficient attention is paid to this important aspect. The costs associated with metabolic dysfunction-associated steatohepatitis (MASH, formerly known as non-alcoholic steatohepatitis) in the US reached 223 billion US dollars in 2017, presenting a significant economic burden (14). This issue affects the recovery rate of work ability and needs further investigation.

MASLD/MAFLD is viewed as a “silent epidemic” characterized by severe difficulties in timely detection and prompt treatment. Currently, approximately 60% of patients have not shown any symptoms during their disease course. One-third of them have already suffered from significant liver fibrosis. Because diagnostic facilities are limited, the missed diagnosis rate exceeds 90% (15, 16). The consecutive gaps indicate room for improvement. The 2020 international expert consensus added some diseases to the diagnostic criteria, including a positive diagnosis of metabolic disorders (17). However, this disease presents no evident early signs, increasing the time to diagnosis (18). The current MASLD/MAFLD prevention and control system focuses on improving metabolism via the scientific dietary guidance of nutrition adjustment and reduced caloric intake. Frequent exercise and dietary modification are the primary basic strategies recommended by leading international authorities (19). The traditional belief holding that vegetarians are healthier (20) is currently being verified in many studies. However, some patients who have been strict vegetarians may still develop progressive MASLD/MAFLD (21). They cannot treat their disease by simply following a healthy diet. Thus, the recognition of multiple metabolic triggers for MASLD/MAFLD is currently lacking. A number of patients still hold the misconception that only the complete avoidance of meat can reduce their risk of developing liver problems.

### 1.1 Review approach

This critical narrative review adopts a purposive, non-systematic approach to examine the apparent paradox whereby certain vegetarian dietary patterns do not provide the expected hepatoprotection against MASLD/MAFLD. Representative studies were identified through targeted searches of PubMed, Web of Science, and Scopus using keywords related to MASLD/MAFLD pathogenesis, vegetarian and plant-based dietary patterns, hepatic lipid metabolism, gut microbiota, and precision nutrition, supplemented by snowball sampling of seminal articles and consensus statements. High-impact original research, meta-analyses, and authoritative reviews published primarily within the past two decades were preferred.

This manuscript does not attempt a systematic or quantitative evidence synthesis, being consistent with the conventions of critical narrative reviewing. Instead, it offers a critically qualified interpretive framework in which mechanistic pathways are presented with explicit evidentiary stratification. The review consistently distinguishes direct human trial evidence, associative observations, biologically plausible mechanisms from experimental models, and hypotheses requiring prospective validation in vegetarian cohorts. Extrapolation from non-vegetarian studies is explicitly acknowledged as conditional when evidence specific to vegetarian populations is unavailable. Therefore, the conclusions should be regarded as interpretive and hypothesis-generating rather than definitive.

## 2 Vegetarian dietary risks and MASLD/MAFLD: traditional perceptions and contemporary paradoxes

### 2.1 Traditional perceptions: hepatoprotective effects of vegetarian diets in MASLD/MAFLD prevention and treatment

The term “vegetarian” encompasses a family of plant-predominant dietary patterns ranging from strict veganism to ovo-lacto-vegetarian, pescatarian, and flexible approaches, with their nutritional profiles varying far more than their shared labels suggest. However, despite the shared emphasis on plant-derived foods, these subtypes differ substantially in nutritional quality, specifically in protein completeness, choline and vitamin B12 status, EPA/DHA availability, and glycemic load—factors that critically determine their respective hepatoprotective efficacy and metabolic risks (20, 22). Generally, plant-based vegetarian diets are believed to decrease MASLD/MAFLD risks through multiple mechanisms. Many polyphenols present at a high concentration in plant-based foods exhibit multiple positive effects by inhibiting the activation of liver inflammatory signals and enhancing the endogenous antioxidant defense system (23, 24). Additionally, they suppress the synthesis of proinflammatory mediators such as tumor necrosis factor-alpha (TNF-*α*) and interleukin-6 (IL-6) at the molecular level through the inhibition of the Toll-like receptor 4 (TLR4)-myeloid differentiation factor 88 (MyD88)-IκB kinase (IKKβ) signal pathway to block nuclear factor kappa-B (NF-κB) nuclear translocation and reduce hepatic inflammation (25). Concurrently, these polyphenols activate the nuclear factor erythroid-2-related factor 2 (Nrf2)/heme oxygenase-1 (HO-1) signaling pathway to increase the activity of the antioxidant defense system, reduce oxidative stress injury of hepatocytes, and inhibit the formation of a positive feedback loop involving c-Jun N-terminal kinase (JNK)/activator protein-1 (AP-1) (24, 26). These pathways work in concert to efficiently regulate hepatic inflammation and oxidative stress, providing a critical molecular basis for improving the status of liver disease, such as in MASLD/MAFLD. Dietary fiber-enriched gut microbiome increases short-chain fatty acids (SCFAs), especially butyrate, and activates the adenosine monophosphate-activated protein kinase (AMPK)/peroxisome proliferator-activated receptor gamma coactivator 1α (PGC-1α) pathway. Thus, hepatocytes promote fatty acid *β*-oxidation efficiency and reduce hepatic triglyceride (TG) levels (27). Furthermore, a plant-based diet falls under the group of effective interventions for MASLD/MAFLD prevention since it has a low energy density and helps with weight loss (Table 1).

**Table 1:** Classification of vegetarian dietary patterns and plant-based diet quality indices in relation to MASLD/MAFLD risk.

Dietary pattern	Description	Key nutritional characteristics	MASLD/MAFLD risk implication	References
Strict vegan	Excludes all animal products (meat, fish, dairy, eggs, honey).	High fiber, polyphenols, and phytochemicals; potential deficiencies in vitamin B12, choline, EPA/DHA, and complete protein; typically low in saturated fat.	Risk is highly diet-quality dependent: whole-food vegan diets may be protective, whereas refined or ultra-processed vegan diets may increase hepatic steatosis and metabolic risk.	Lv et al. (22), Wang et al. (26), and Li et al. (28)
Lacto-ovo vegetarian	Excludes meat and fish; includes dairy and eggs.	Moderate protein quality (eggs provide complete protein); dairy offers preformed vitamin B12 and calcium; risk of excess saturated fat from dairy if not managed.	Generally favorable lipid profile; adequate choline and B12 from eggs reduce genetic susceptibility risk (PNPLA3-choline interaction); evidence for MASLD/MAFLD protection is heterogeneous.	Wang et al. (26), Herreman et al. (35), Luukkonen et al. (60), and Wang et al. (61)
Pescatarian	Excludes meat and poultry; includes fish, dairy, and eggs.	Excellent source of preformed EPA/DHA from fish; complete protein from fish and eggs; low risk of essential fatty acid deficiency.	Favorable n-6:n-3 fatty acid ratio due to fish intake; lowest MASLD/MAFLD risk among vegetarian subtypes; EPA/DHA may attenuate inflammation and hepatic fat accumulation.	Mäkelä et al. (32) and Dempsey et al. (43)
Flexitarian	Primarily plant-based with occasional meat/fish consumption.	Flexible macronutrient profile; allows strategic inclusion of high-quality animal protein and micronutrients while maintaining predominantly plant-based intake.	Risk profile similar to high-quality plant-based diets; occasional fish/meat consumption may mitigate B12, choline, and EPA/DHA deficiencies without requiring systematic supplementation.	Paternostro et al. (20) and Lv et al. (22)
Healthy plant-based (hPDI)	Emphasizes whole grains, legumes, nuts, vegetables, fruits, and healthy plant oils; minimizes refined carbohydrates and added sugars.	High in fiber, polyphenols, and antioxidants; legume-grain complementarity supports protein adequacy; flaxseeds/walnuts may optimize the n-6:n-3 ratio; prebiotic fiber promotes gut microbial diversity.	Inverse association with metabolic syndrome and hepatic steatosis in prospective cohort studies; low glycemic load may inhibit ChREBP signaling; enrichment of butyrate-producing microbiota.	Lv et al. (22), Li et al. (28), Livingston et al. (87), Ni et al. (88), Zhang et al. (89), Agrinier et al. (90), and Rahimlou et al. (91)
Unhealthy plant-based (uPDI)	High intake of refined grains, sweets, sugar-sweetened beverages, fruit juices, ultra-processed vegetarian products, and added sugars; excludes animal products.	Low fiber, high glycemic load; incomplete protein (low lysine/methionine); imbalanced n-6:n-3 ratio (>15:1) from vegetable oils; high fructose intake; low micronutrient density.	Highest MASLD/MAFLD risk among vegetarian subtypes; ChREBP-mediated DNL activation; impaired ApoB100–VLDL assembly; TLR4–NF-κB inflammatory pathway activation; may confer metabolic risk comparable to or exceeding omnivorous diets.	Li et al. (28), Régnier et al. (34), Liput et al. (42), Hall et al. (58), Grinshpan et al. (108), and Henney et al. (109)

### 2.2 Current paradox: the contradiction between traditional dietary stereotypes and the reality of MASLD/MAFLD

The beliefs and traditions related to the decrease in MASLD/MAFLD risks by vegetarian diets have existed for a long time. However, many high-quality recent studies have demonstrated that the MASLD/MAFLD rate of vegetarians is not as low as that of non-vegetarians. Large-scale epidemiological studies by the US National Health and Nutrition Examination Survey (NHANES) have not found any connection between vegetarians and MASLD/MAFLD after controlling for factors such as body mass index (BMI) and diabetes mellitus (28). Furthermore, recent meta-analyses have shown that MASLD/MAFLD prevalence among vegetarian patients is approximately 22–28.6%, although they did not statistically compare the data to the rate of meat-eating people (29, 30). In total, the above-converged evidence shows that vegetarians’ diet alone cannot reduce the MASLD/MAFLD rate worldwide. On its own, it requires global dietary structure improvements and metabolic parameter adjustments to mitigate MASLD/MAFLD. Remarkably, the mechanistic understanding obtained from a carefully matched research design of young Chinese adults showed that vegetarians exhibited elevated levels of inflammatory factors TNF-*α* and IL-6 (21). These inflammatory factors are involved in liver steatosis and non-alcoholic hepatitis pathogenesis by increasing insulin resistance and lipotoxicity (25). Thus, it provides a molecular basis for explaining the inability of a monolithic vegetarian diet to prevent the MASLD/MAFLD progression, highlighting the contradiction regarding liver protection in the traditional vegetarian management of MASLD/MAFLD.

## 3 Deconstruction of the vegetarian hepatoprotection paradox: a three-tier cascade including upstream dietary insults, genetic amplifications, and downstream microbiome entrainment

A proposed framework for understanding the “vegetarian hepatoprotection paradox” in MASLD/MAFLD posits three interacting dimensions. Dietary factors may constitute an upstream layer of metabolic perturbation, which could be amplified by host genetic variability at an intermediate level and further modulated by gut microbiota configurations downstream. Each of these dimensions is examined in detail below, focusing on the distinction between direct evidence, associative observations, biologically plausible mechanisms, and hypotheses requiring further validation.

### 3.1 Risk dietary patterns and structural deficiencies in related vegetarian diets

At the upstream tier, i.e., dietary factors, the MASLD/MAFLD risk associated with certain plant-based diets at this level does not stem from a whole-grain diet but from four structurally deficient subpatterns that may independently impair hepatocellular lipid processing. For example, a high-refined carbohydrate-low-protein pattern (predominated by refined white rice and flour) is lacking in an abundant supply of high-quality plant proteins (31). An n-6 fatty acid–dominated pattern (the persistent use of a large amount of long-chain omega-6 vegetable oil) may contribute to hepatic inflammation when n-3 fatty acid availability is limited and the overall lipid environment favors the production of proinflammatory mediators (32). The high-fructose-little-fiber pattern (the excessive intake of fruit juice, sugar-sweetened beverages, and sweetened processed products) was proposed as a factor that may affect hepatic fructose metabolism and intestinal barrier integrity (33). A severely processed plant-based dependence type also exists, in which the frequent consumption of products causing hyperglycemia may perturb the intestinal carbohydrate response element binding protein (ChREBP)-solute carrier family 2 (facilitated glucose/fructose transporter), member 5 (SLC2A5) axis and contribute to alterations in the gut-liver axis (34). Together, they are indirectly linked to the MASLD/MAFLD progression associated with particular dietary deficiencies in plant-based diets. Thus, carefully designing a dietary survey for vegetarians in clinical practice is important to help them correct such deviations and prevent the excessive intake of certain foods due to a lack of understanding.

#### 3.1.1 Highly refined carbohydrate–low protein pattern: mediation of hepatic lipid accumulation by the amino acid deficiency-ApoB100-VLDL pathway

The diet with the highly refined carbohydrate–low protein pattern has a prominent excess of refined staple foods such as white rice and refined wheat products, as well as an inadequate amount of high-quality plant-based proteins, which provide insufficient amounts of essential amino acids (35). Severe protein deficiency is a recognized cause of hepatic steatosis in humans, as classically observed in kwashiorkor, in which impaired very low-density lipoprotein (VLDL)-dependent lipid export serves as a contributing mechanism (36). However, stable isotope studies in children with severe acute malnutrition showed that the VLDL apolipoprotein B-100 (ApoB100) synthesis rate is not necessarily reduced (37), suggesting that the ApoB100-VLDL pathway in humans is more complex than in rodent models. Compared with animal protein sources, most plant-based protein sources provide a significantly lower amount of amino acids (35). Notably, lysine and methionine are particularly limited. From the perspective of molecular pathology, the synthesis rate of ApoB100, one of the major structural components within VLDL, may be influenced by the insufficient amount of these two essential amino acids (38). Limiting its synthesis may thereby contribute to VLDL assembly and secretion abnormalities, which may hinder the normal release of newly synthesized hepatic triglycerides. These, in turn, accumulate in hepatocytes under conditions of sustained substrate excess (39, 40). This proposed ApoB100-VLDL mechanism provides a plausible, although not definitively established, molecular explanation regarding the hepatosteatosis risk in the context of a high-refined-carbohydrate, low-protein vegetarian pattern. This pathway was primarily characterized in experimental models of severe protein and choline deficiency with poor amino acid complementarity; hence, its direct relevance to free-living vegetarian populations with MASLD/MAFLD remains to be validated through prospective clinical studies. The specific conditions under which this mechanism may become clinically relevant (i.e., severe protein deficiency, suboptimal amino acid complementation across protein sources, and concurrent low choline intake) should be acknowledged as potentially applicable only to a subset of vegetarian dietary patterns.

#### 3.1.2 The n-6/n-3 ratio in plant-based diets: re-evaluating its role in hepatic inflammation and fibrosis amidst metabolic and dietary contexts

In a vegetarian diet heavily relying on soybean oil (n-6/n-3 = 7–50:1), its large ratio of n-6 to n-3 fatty acids (>15:1) is ascribed to differences in dietary sources of these two fatty acids (41), with linoleic acid accounting for more than half as part of common vegetable oils such as soybean and corn oils (42). Meanwhile, α-linolenic acid and eicosapentaenoic acid (EPA)/docosahexaenoic acid (DHA) mainly come from flaxseed oil and cold-water fish, respectively. Strict vegetarians avoiding the latter may have a reduced intake of preformed n-3 fatty acids (43).

Whether this fatty acid imbalance directly promotes hepatic pathology may depend on several contextual factors, including the overall lipid quantity, concurrent EPA/DHA availability, the specific food sources of n-6 fatty acids, and individual metabolic background. An excess of n-6 polyunsaturated fatty acids undergoes metabolic reactions to produce arachidonic acid (AA), which further generates bioactive mediators, including leukotriene B4 (LTB4) and prostaglandin E2 (PGE2). However, n-6 fatty acids, including linoleic acid and arachidonic acid, also serve essential physiological functions and are not inherently hepatotoxic. Their metabolic fate is context-dependent, influenced by the available enzymatic machinery, competing substrates (notably n-3 fatty acids), and the overall inflammatory milieu. The activated LTB4 and PGE2 may promote the production of proinflammatory factors in Kupffer cells via the TLR4-NF-κB signaling pathway (44, 45). This represents one biologically plausible pathway that may contribute to hepatic inflammation. However, caution should be applied to extrapolating this molecular mechanism to the progression of liver fibrosis in vegetarian populations. The relationship between n-6/n-3 ratios and hepatic outcomes is unlikely to be linear because it is modulated by the total fat intake, the presence of EPA- and DHA-derived compensatory anti-inflammatory lipid mediators, individual genetic variation in fatty acid desaturation and elongation enzymes, and the overall dietary pattern. Current evidence does not support a simple model in which higher n-6/n-3 ratios directly cause liver inflammation and fibrosis in vegetarian individuals.

#### 3.1.3 High-fructose diet: neogenesis and hepatic injury driven by fructose metabolic bypass and gut-liver axis dysregulation

The high-fructose diet is generally linked to the increased consumption of fructose and fructose-containing products. While the diet of vegetarians typically contains more fructose, distinguishing between whole fruit consumption and intake of fructose-dense processed products is essential. The adverse hepatic effects of fructose were primarily documented in the context of sugar-sweetened beverages, fruit juices, refined carbohydrate products, sugar-dense dried fruits, and added sugars—not whole, fiber-rich fruits containing polyphenols and other bioactive compounds. Whole fruits, by virtue of their fiber content and polyphenol composition, may attenuate fructose absorption and modify its metabolic fate; therefore, they should not be implied to be inherently hepatotoxic. Additionally, their metabolic pathway includes an exception for the regulating system, preventing the excessive accumulation of liver fat. Fructose does not require insulin to promote its oxidation in the body, bypassing phosphofructokinase-1 (PFK-1), and lacks a negative regulatory mechanism (46). Fructose is absorbed into the liver rapidly after entering it due to an efficient glucose transporter 5 (GLUT5) carrier in hepatocytes. Within hepatocytes, fructose can be immediately phosphorylated by ketohexokinase (KHK). Afterward, it is directly converted into substrates for TG synthesis to drive *de novo* lipogenesis (DNL) within the cell (47). The basic mechanism is that fructose metabolism activates the transcription factor ChREBP to cause “synthesis-deposition” without restraint from energy-sensing pathways such as AMPK (48, 49). However, this mechanism was characterized primarily in models of high-fructose beverage consumption and may not apply equivalently to fructose consumed as part of whole fruits.

Moreover, fructose may contribute to hepatic injury through pathways involving the dysfunction of the gut-liver axis. Morphologically, fructose impairs the function of the intestinal barrier, causing changes in the microbiota community structure that increase lipopolysaccharide (LPS) and endogenous ethanol in the gut lumen (50–52). Then, microorganism-associated metabolites may traverse the compromised epithelial barrier, reach the portal venous circulation, and enter the liver. LPS in the hepatic microenvironment activates TLR4 on Kupffer cells to trigger the NF-κB-mediated production of proinflammatory cytokines and induce oxidative stress through the formation of cytochrome P450 2E1-mediated reactive oxygen species. These converged pathways synergistically enhance hepatocellular necroinflammation (50, 52). As noted above, the experimental and epidemiological evidence supporting this gut-liver axis mechanism derives predominantly from studies of refined fructose sources, particularly sugar-sweetened beverages, rather than whole fruit consumption.

In the meantime, the reduction in enterocyte-specific Vanin-1 (VNN1) enzyme activity after fructose binding decreases bile acid–methylcysteamine (BA-MCY) levels and impairs farnesoid X receptor (FXR) sensitivity. Subsequently, this signaling defect spreads to the liver through the enterohepatic recirculation of BAs. Thus, it continuously enhances enzyme expression related to lipid synthesis and exacerbates fatty liver disease (53).

#### 3.1.4 Abnormal regulatory pattern of high blood sugar vegetarian diet: the joint promotion of the over-activation of the ChREBP pathway and the disruption of the gut-liver axis

The main characteristic of the high blood sugar vegetarian diet is the long-term consumption of refined foods with a high glycemic index (GI), such as white bread, white rice, and refined noodles. High-sugar fruits include red dates, sugarcane, and lychees (54). The central metabolic dysfunction is caused by the stimulation of activated ChREBP through the *de novo* lipid synthesis pathway mediated by protein phosphatase 2A (PP2A), which increases blood glucose levels after high-GI vegetable meals (55). Intracellular ChREBP combines with Max-like protein X (MLX) in a dimer form and binds to the promoter region of lipid synthesis–related target genes. Hence, the threefold rise in hepatic *de novo* lipogenesis (DNL) results in the synthesis and accumulation of excessive TGs within hepatocytes (56).

This mechanism basically explains the clinical phenomenon of the “vegetarian hepatoprotection paradox.” Due to increased hepatic lipids from the defective hypothalamic leptin signaling, 28.6% of the MASLD/MAFLD group had a “leptin-resistant” phenotype with a normal BMI (54). These people maintain a “normal body weight” through vegetarianism; however, they eat high-GI foods that constantly activate the ChREBP-Slc2a5 axis to induce concealed hepatic lipid deposition. Consequently, the “vegetarian liver protection” strategy may fail to confer the expected protection (57).

The long-term consumption of refined, high-GI products, such as white bread and refined noodles, and high-sugar fruits, such as red dates, sugarcane, and lychees, will quickly increase postprandial blood sugar levels and cause the release of a large amount of insulin. Such a metabolic disorder is activated by promoting the ChREBP-mediated DNL pathway because glucose derivatives from high-GI vegetative foods can induce PP2A to deactivate protein phosphorylation, which thereby deactivates ChREBP and induces its nuclear translocation (34). Intranuclear ChREBP forms a functional dimer with MLX and binds to the promoter of lipid synthesis target genes. This results in a threefold increase in the rate of hepatic DNL, producing and accumulating excessive TGs inside liver cells (58, 59).

### 3.2 Mitigation of the genetic trap by vegetarian diets: hepatic lipid crisis amplified by gene-nutrient interactions

At the middle level, genetic enhancement determines the susceptibility to MASLD/MAFLD, which depends on the genotype and environmental factors introduced earlier.

#### 3.2.1 PNPLA3 I148M variant: low-choline and high-fructose amplification

The combination of the PNPLA3 I148M variant and the inherent lower choline content causes insufficient hepatic phosphatidylcholine synthesis and defective VLDL assembly (60). Consequently, this variant reduces TG hydrolase activity by modifying liposome surfaces. This forms a “phospholipid synthesis–liposome hydrolase” double-barrier system (61). Then, a rise in fructose will intensify liver lipogenesis (62). Through a combination of genes and nutrients, this increased risk of liver fat accumulation is estimated to be 3–4 times higher among vegetarian carriers compared with intact amino acid-choline–containing omnivores (60).

#### 3.2.2 TM6SF2 E167K variant: high-refined-carbohydrate and incomplete-protein amplification

The TM6SF2 E167K variant connects to some common issues observed in traditional vegetarian dietary patterns. First, highly pure carbohydrates from plants play a role in triggering hepatic DNL to increase TG substrate content (63). Second, plants have insufficient amounts of protein components, such as methionine and lysine, for ApoB100 production, which undermines the formation stability of VLDL particles (64, 65). The TM6SF2 E167K mutant disrupts the lipid metabolism pathway of VLDL as well. The three converging deficiencies generate a “three-blockage” effect at the level of liver TG export, especially in vegetarians. They are characterized by increased TG substrate levels due to refined carbohydrates, insufficient ApoB100 carriers owing to inadequate plant protein intake, and the impairment of lipidation machinery. Omnivorous diets providing a complete source of animal proteins and low GI have a significantly reduced risk via preserved ApoB100-dependent VLDL assembly.

### 3.3 Microbiota dysregulation in the gut-liver axis: the cascading amplification of vegetarian metabolic defects

**Figure 1:**
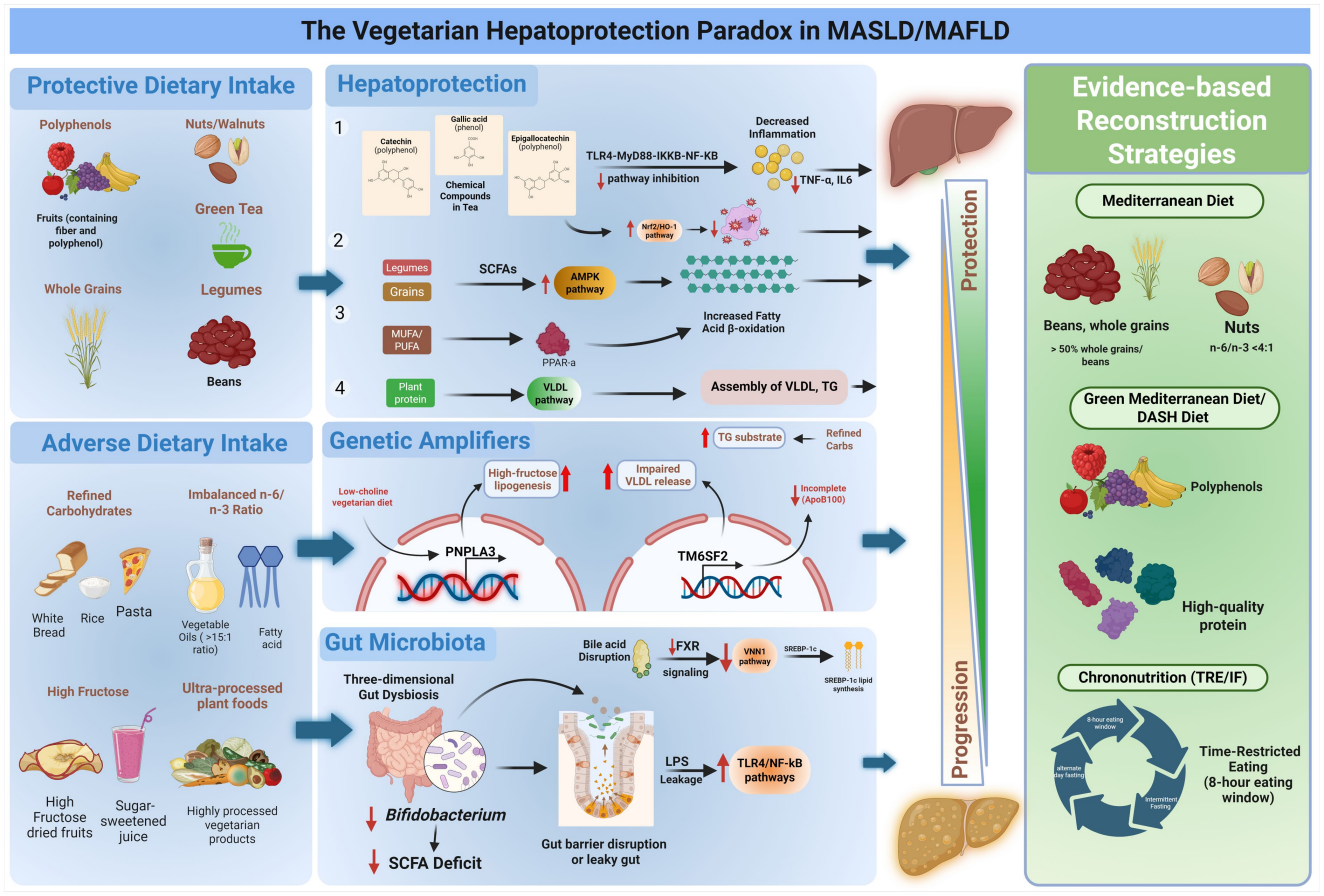
Schematic of the “vegetarian hepatoprotection paradox” in MASLD/MAFLD. (Upper left) Protective dietary intake—polyphenol-rich fruits and tea, nuts, whole grains, and legumes—confers hepatoprotection through four convergent mechanisms: (1) TLR4-MyD88-IKK*β*-NF-κB pathway inhibition; (2) SCFA-mediated AMPK activation promoting fatty acid β-oxidation; (3) PPAR-*α* stimulation by MUFAs/PUFAs; and (4) plant protein-dependent VLDL-TG assembly. (Lower left) Four structurally deficient subpatterns—high-refined-carbohydrate/low-protein (white bread, rice, pasta), imbalanced n-6:n-3 ratio (>15:1 vegetable oils), high-fructose (juice, dried fruits), and ultra-processed plant foods—independently impair hepatocellular lipid processing. (Center) PNPLA3 I148M and TM6SF2 E167K variants function as genetic amplifiers: the former intensifies lipogenesis under low-choline/high-fructose conditions, while the latter impairs VLDL release amid refined carbohydrates and incomplete protein. Three-dimensional gut dysbiosis—SCFA deficit, LPS leakage, and bile acid disruption—propagates inflammation via TLR4/NF-κB and FXR/VNN1 pathways. (Right) Evidence-based reconstruction strategies include Mediterranean diet (>50% whole grains/beans, n-6:n-3 < 4:1), Green Mediterranean/DASH (polyphenol-rich), high-quality plant-based protein (complementary amino acids), and chrononutrition (8-h TRE).

At the downstream tier, namely the microbiology-mediated entrapment, gut microbes may function as convergent executors to transform upstream dietary and genetic deficits into a self-sustaining vicious cycle of hepatobiliary inflammation and lipid homeostasis. When diets mainly contain high amounts of refined carbohydrates and fructose, the gut microbiota structure is significantly changed: the number of butyrate-producing bacteria dramatically decreases. This leads to the synthesis of insufficient SCFAs (66), disrupts the negative feedback mechanism on the ChREBP pathway, and results in an about twofold increase in liver DNL. Enriched Gram-negative bacteria produce more LPS and endogenous ethanol due to overstimulation (67). Through entry into the liver via the portal vein, the activation of TLR4/NF-κB inflammasomes acts synergistically with an overabundance of n-6:n-3 ratio (68, 69). Crucially, the changed gut-BA metabolism inactivates the FXR signal pathway (70). Together with the damaged VNN1-pathway response, this eliminates the repression of the sterol regulatory element-binding protein (SREBP)-1c-lipid biosynthesis program. The three-dimensional dysbiosis of “SCFA deficit–leakage of LPS–BA disorder” may be greatly aggravated in the presence of certain genetic variants. This leads to the self-reinforcing cycle of “structural defects–dysbiosis-high production–output block chronic inflammation” hepatic lipolysis in the vegetarian population (Table 2; Figure 1).

**Table 2:** Evidence hierarchy for mechanistic pathways linking vegetarian dietary subpatterns to MASLD/MAFLD risk.

Pathway	Representative dietary exposure	Evidence type	Directness to vegetarian MASLD/MAFLD populations	Certainty level	Clinical implication	References
High-GI carbohydrate–ChREBP-mediated de novo lipogenesis.	White rice, white bread, refined noodles, refined cereals, high-GI vegetarian staples, frequent intake of refined starches.	RCTs of glycemic modification, metabolic feeding studies, and mechanistic studies of ChREBP activation and hepatic DNL.	Indirect to moderate. Most trials were conducted in mixed-diet populations, but the exposure pattern is highly relevant to refined vegetarian diets.	High	High-GI carbohydrate restriction is supported by Level I evidence; particularly relevant for vegetarian diets reliant on refined staples.	Régnier et al. (34), Vanegas et al. (56), Agius et al. (57), Hall et al. (58), and Yu et al. (59)
n-6:n-3 fatty acid imbalance and inflammatory lipid signaling.	High intake of linoleic acid–rich vegetable oils such as soybean and corn oils; insufficient preformed EPA/DHA intake; high n-6/n-3 ratio.	Mechanistic studies of fatty acid metabolism and inflammatory mediators; biomarker and observational studies.	Indirect. Limited direct evidence on liver outcomes in vegetarian MASLD/MAFLD populations.	Moderate	The clinical goal is not to eliminate essential n-6 fatty acids, but to improve overall dietary fat quality; particularly in strict vegetarians, increased intake of flaxseeds, chia seeds, walnuts, and algae-derived DHA/EPA may be considered.	Simopoulos et al. (41), Liput et al. (42), Dempsey et al. (43), Gao et al. (44), and Torrissen et al. (45)
Fructose-driven hepatic de novo lipogenesis.	Sugar-sweetened beverages, fruit juices, refined fructose-rich products, high-fructose corn syrup (>25 g/day).	Controlled feeding trials, isotope tracer studies, meta-analyses, and epidemiological studies in general populations.	Indirect. Limited vegetarian-specific MASLD/MAFLD evidence.	Moderate	Limiting refined fructose is strongly supported across dietary patterns, including vegetarian diets. Whole fruit consumption should be distinguished from processed fructose sources.	Ramirez et al. (46), Fowle-Grider et al. (47), Baharuddin et al. (48), and Gugliucci et al. (49)
High-fructose–related gut–liver axis injury.	Long-term intake of refined fructose, sugar-sweetened beverages, fruit juice, and sweetened processed foods.	Gut–liver axis mechanistic studies; animal experiments; epidemiological and biomarker studies.	Indirect. Evidence from high-fructose models, with limited extrapolation to whole fruit intake.	Moderate	In vegetarian MASLD/MAFLD patients, clinicians should distinguish “high fruit intake” from “high liquid sugar/added sugar intake”; priority should be given to reducing liquid sugars and ultra-processed sweet foods.	Li et al. (50), Cho et al. (51), and Deng et al. (52)
Refined-carbohydrate/fructose-driven dysbiosis: SCFA deficiency and weakened negative feedback on ChREBP.	Plant-based diets dominated by refined carbohydrates, fructose, and low-fiber processed foods.	Gut microbiota mechanistic evidence; SCFA metabolism evidence; ChREBP–DNL regulatory evidence.	Moderately indirect. Dysbiosis is associated with MASLD, but the causal chain in vegetarian subtypes still requires longitudinal studies.	Moderate	“Plant-based” does not necessarily mean “high fiber.” Whole grains, legumes, vegetables, nuts, and whole fruits should be increased, while refined carbohydrates should be reduced.	Qin et al. (66)
LPS/endogenous ethanol–TLR4–NF-κB inflammatory amplification.	High-sugar, high-refined-carbohydrate, low-fiber diets; may coexist with a high n-6/low n-3 lipid environment.	Microbial endotoxin evidence; portal-vein inflammation mechanism evidence; TLR4/NF-κB activation evidence.	Indirect. The gut–liver axis mechanism is clearly relevant to MASLD, but evidence specific to vegetarian populations is limited.	Moderate	Clinical management should correct high sugar, high GI, low fiber, and fatty-acid imbalance simultaneously, rather than focusing only on meat avoidance.	Stamation et al. (67), Schnabl et al. (68), and Yang et al. (69)
Bile acid–FXR/SREBP-1c dysregulation.	Plant-based diets dominated by refined carbohydrates and fructose, with low fiber intake and reduced microbial diversity.	Bile acid metabolism evidence; FXR signaling mechanism evidence; SREBP-1c lipogenic program evidence.	Indirect. Mechanistically plausible, but population-level causality and vegetarian specificity remain to be verified.	Moderate	The gut microbiome and bile acid metabolism may be considered potential intervention targets. Currently, the more practical approach is to increase dietary fiber and the proportion of whole foods.	Wu et al. (70)
Amino acid deficiency–ApoB100–VLDL lipid export impairment pathway.	Low-quality plant proteins deficient in lysine and methionine; inadequate protein complementation; severe protein deficiency.	Human observations in severe protein deficiency; kwashiorkor studies; stable isotope studies; animal and mechanistic studies.	Indirect. Limited direct evidence in free-living vegetarian MASLD/MAFLD populations.	Low to moderate	Severe protein deficiency is a recognized cause of hepatic steatosis, but the ApoB100–VLDL mechanism remains incompletely validated in typical vegetarian populations.	Herreman et al. (35), Shan et al. (36), Badaloo et al. (37), Chen et al. (38), Fisher et al. (39), and Berndsen et al. (40)
PNPLA3 I148M × low choline and high fructose exposure.	Low choline intake; insufficient phosphatidylcholine precursors; high-fructose diet; increased sugar-sweetened beverage intake.	Genetic association studies, gene–diet interaction analyses, and *in vitro* functional studies.	Very indirect. No prospective intervention studies have specifically tested this interaction in genotyped vegetarian MASLD/MAFLD cohorts.	Low to moderate	Biologically plausible but speculative; choline supplementation (or egg consumption for ovo-vegetarians) may be considered for PNPLA3 I148M carriers following strict vegan diets.	Luukkonen et al. (60), Wang et al. (61), and Zhao et al. (62)
Fructose–VNN1–BA-MCY–FXR pathway.	Long-term high-fructose intake, especially from refined fructose sources.	Molecular mechanistic studies; animal model evidence; limited human translational evidence.	Indirect. Limited direct evidence in vegetarian MASLD/MAFLD populations.	Low	This pathway may help explain fructose-related gut–liver metabolic disruption, but should not currently be used as a standalone basis for clinical decision-making.	Hernandez et al. (53)
TM6SF2 E167K × refined carbohydrate and incomplete protein exposure.	High refined-carbohydrate intake; high-GI staple foods; insufficient plant-protein quality; relative methionine/lysine deficiency.	Genetic association studies, human metabolic studies, and in vitro evidence related to VLDL lipidation and triglyceride export.	Very indirect. Current evidence is extrapolated from genetic and metabolic studies rather than vegetarian-specific clinical trials.	Low	Hypothesis-generating only; no clinical trial evidence supports genotype-specific dietary modification for TM6SF2 variants in vegetarian patients.	Ehrhardt et al. (63), Borén et al. (64), and Kim et al. (65)

## 4 Dietary pattern reconstruction: from the “vegetarian hepatoprotection paradox” to evidence-based precision nutrition

According to the above-presented pathogenesis analysis, solving the “vegetarian hepatoprotection paradox” needs to stay apart from a simple “vegetarian” concept and instead guide dietary pattern reconstruction based on metabolic advantages (18). The MD, the DASH diet, TRE, and high-quality plant-based diets all have sufficient level-I evidence to support them to break through the double-sided contradiction of vegetarian diets enhancing the efficiency of metabolism in the future (71). All of them ensure high-quality proteins, optimize fatty acid composition, and control the glycemic load of carbohydrates to regulate the function of the intestinal–liver axis (19).

### 4.1 MD: the gold standard for MASLD/MAFLD dietary intervention

The MD underlies the evidence-based approach to the dietary management of MASLD/MAFLD. It has been universally recommended by international guidelines, such as the European Association for the Study of the Liver (EASL), the European Association for the Study of Diabetes (EASD), the European Association for the Study of Obesity (EASO), and the American Gastroenterological Association (AGA) (72). A meta-analysis and systematic review of 37 randomized controlled trials showed that the MD significantly decreased body weight (weighted mean difference [WMD] = −2.38 kg, 95% confidence interval [CI]: −4.11–−0.66), BMI (WMD = −0.70 kg/m^2^, 95% CI: −1.03–−0.36), waist circumference (WMD = −1.56 cm, 95% CI: −3.02–−0.09), and alanine aminotransferase (ALT) levels (WMD = −3.96 IU/L, 95% CI: −6.54–−1.38) compared with control diets (73). The benefits are anthropometric and hepatic, thereby having hepatoprotective effects. These effects are provided by multi-system metabolic reprogramming comprising adipose tissue regulation, visceral fat reduction, and direct liver action improvement.

The MD includes >50% of fresh fruits, vegetables, and cereals while containing less fat, including animal fats such as butter, cheese, duck eggs, and pork belly (74). The MD is not a true vegetarian diet; it rather includes fish and dairy products to compensate for the lack of protein in a pure plant-based diet (74). These distinct nutritional characteristics contribute to hepatoprotective effects through multiple targets: monounsaturated fatty acids (MUFAs) and polyunsaturated fatty acids (PUFAs) can activate the peroxisome proliferator-activated receptor-*α* (PPAR-α) pathway to promote fatty acid *β*-oxidation (74). Dietary fiber increases the production of SCFAs and improves the function of the gut–liver axis. Polyphenolic compounds reduce hepatic inflammation by blocking the TLR4/NF-κB signal pathway (75).

Thus, the “green MD” adds a higher proportion of plants and increases its beneficial effects more significantly than the original MD. In the DIRECT–PLUS randomized controlled trial (*n* = 294; follow-up after 18 months), a daily supplementary dose of 28 g walnuts, 3–4 cups of green tea, and 100 g of Mankai were added to the standard MD system, including low reds/processed meats. This achieved an intrahepatic fat decrease rate of 38.9%, which was more significant than 19.6% for the conventional MD or 12.2% for the healthy dietary guideline group. MASLD/MAFLD incidence fell from 62 to 31.5% (72). The enhanced effect was positively associated with plasma polyphenols and increased microbial β-diversity. Therefore, the combined application of polyphenol-rich and plant protein–dense foods strengthened the hepatoprotective effect of the MD.

### 4.2 DASH diet: dual cardiovascular-hepatic metabolic effects

DASH refers to an increase in vegetables, fruit, whole grain foods, and legumes but limits the intake of red meat and processed food. As another evidence-based dietary pattern, the DASH diet demonstrates complementary clinical value to the MD in MASLD/MAFLD management. Experimental evidence indicated that the DASH diet group showed a greater reduction in liver steatosis score, ALT, aspartate aminotransferase-to-platelet ratio index (APRI), and lipoprotein accumulation score compared with the low-calorie control group (*p* < 0.05) (76). Notably, the better improvement in the liver status exhibited no statistical differences in group size. Therefore, hepatoprotective effects exceeded pure caloric reduction (77). Sangouni et al. performed a randomized controlled trial using the DASH diet to reduce liver fibrosis and non-alcoholic steatohepatitis (78).

The dietary habits of these people can substantially reduce the risk of metabolic syndrome by multiple pathways. High potassium and magnesium intake improves vascular endothelial function and insulin sensitivity. Furthermore, dietary fiber promotes the proliferation of probiotics and optimizes the production of SCFAs. Additionally, low GI carbohydrate levels prevent the excessive activation of the ChREBP pathway (77). Therefore, the DASH diet can help maintain the liver-and-heart-function balance of hypertensive patients or those with MASLD/MAFLD and at high risk of cardiovascular disease (79).

### 4.3 Time-restricted feeding and intermittent fasting (IF): applications of chrononutrition

Chrononutrition studies the effect of changes in diet time or other factors on circadian rhythm–based metabolic health (80, 81). The two ways include TRE and IF, such as the 5:2 or alternate day fasting model. In TRE, the food intake is limited to 6–10 h per day (82).

Some approaches exhibit a hepatoprotective activity during MASLD/MAFLD clinical management. Based on a 12-article systematic review, TRE and IF both improved hepatic steatosis by improving insulin sensitivity and reducing inflammatory factors (80). The 5:2 IF regimen markedly improved liver stiffness, while TRE positively influenced the severity of steatosis (83). These changes are partly unrelated to weight loss. Therefore, feeding time may independently regulate hepatocyte metabolism (84).

Various regulation levels are activated through the AMPK pathway to stimulate fatty acid oxidation and autophagy during fasting. Simultaneously, regular short-term fasting increases gut microbial α-diversity and lowers serum trimethylamine N-oxide (TMAO) concentrations to protect the liver (84). The heterogeneous impacts of different protocols require selection according to the metabolic phenotype and tolerance (85).

### 4.4 High-quality plant-based diets: key to resolving the “vegetarian hepatoprotection paradox”

In fact, this phenomenon can be explained as “it is the quality of vegetables rather than a vegetable-based diet itself.” The following guidelines need to be followed to develop a high-quality plant-based diet: protein quality should be optimized to achieve a complete essential amino acid profile in combination with legumes and whole grain foods such as lentil + brown rice and chickpea + quinoa (86). Balancing fatty acid ratios, replacing soybean oil with flaxseed oil and walnuts to maintain an n-6/n-3 ratio below 4:1, and inhibiting the activation of the ChREBP-SLC2A5 axis are necessary (41, 87). Furthermore, the quality of carbohydrates should be controlled, and whole grains, legumes, and resistant starch should be used for preventing the excessive activation of the ChREBP-Slc2a5 axis (88, 89). Finally, the intake of polyphenols and dietary fiber should be increased (at least 400 g of non-starchy vegetables daily, about 200 g of berry fruit, and approximately 30 g of nuts) to provide adequate polyphenols and prebiotics (90, 91) (Table 3).

**Table 3:** Practical clinical framework for dietary strategies targeting nutritional deficits and adverse dietary factors in MASLD/MAFLD management.

Dietary strategy	Targeted nutritional issue	Core components	Main mechanisms	Evidence type/level	References
Mediterranean diet (MD).	Excess saturated fat intake; low intake of MUFAs/PUFAs, fiber, polyphenols, and whole grains; high intake of refined carbohydrates and red/processed meat.	Olive oil, vegetables, fruits, whole grains, legumes, nuts; moderate fish and dairy; limited red meat.	Improves hepatic metabolism and reduces intrahepatic fat accumulation; promotes fatty acid β-oxidation via PPAR-α activation; increases SCFA production; attenuates oxidative stress and TLR4/NF-κB-mediated inflammation.	Level I (37 RCTs)	Arita et al. (73), Palumbo et al. (74), and Mantovani et al. (75)
Green Mediterranean diet.	Insufficient polyphenol and plant-protein intake; excessive red/processed meat intake; persistent oxidative stress, inflammation, and gut microbiota dysbiosis.	Standard MD + walnuts, green tea, Mankai(a *Wolffia globosa* strain); Reduced red/processed meat intake.	Enhances polyphenol-mediated anti-inflammatory effects; improves gut microbial diversity; may reduce intrahepatic fat more strongly than conventional Mediterranean diet.	Level I (DIRECT-PLUS)	Yaskolka Meir et al. (72)
DASH diet.	High sodium intake; low potassium, magnesium, calcium, fiber, and whole-grain intake; high cardiometabolic risk, hypertension, insulin resistance, or metabolic syndrome.	Fruits, vegetables, whole grains, legumes, nuts; limited sodium, red meat, and added sugars.	Lower blood pressure and cardiometabolic risk; improved insulin sensitivity and endothelial function; increased fiber-driven SCFA production; reduced glycemic load and excessive ChREBP-mediated de novo lipogenesis.	Level I (multiple RCTs)	Badali et al. (76), Sangouni et al. (77), Sangouni et al. (78), and Nilghaz et al. (79)
Time-restricted eating (TRE).	Prolonged eating window; late-night eating; circadian misalignment; excessive evening energy intake; repeated postprandial hyperglycemia and hyperinsulinemia.	Time-restricted eating, commonly 6–10 h eating windows; early time-restricted eating when feasible; 5:2 intermittent fasting or alternate-day fasting in selected patients.	Enhanced circadian alignment, insulin sensitivity, AMPK activation, fatty acid oxidation, and autophagy; reduced hepatic steatosis and liver stiffness; improved gut microbial diversity and decreased inflammatory metabolites such as TMAO.	Level II (emerging RCTs)	Lange et al. (80), Haddad et al. (82), and Zhang et al. (84)
High-quality plant-based diet.	Low protein quality; inadequate essential amino acid complementation; low choline, vitamin B12, iron, zinc, calcium, vitamin D, and preformed omega-3 intake; high refined-carbohydrate, high-GI, high-fructose plant-food dependence.	Whole grains, legumes, nuts, vegetables, fruits; legume-grain protein complementation; flaxseed/walnuts for n-3; n-6:n-3 < 4:1.	Complete amino acid complementation; optimized fatty acid balance / reduced n-6:n-3 ratio; ChREBP–SLC2A5 axis suppression; improved carbohydrate quality with whole grains, legumes and resistant starch; increased polyphenol and prebiotic fiber intake, gut microbiota modulation and metabolic protection.	Level II (cohort + RCT).	Simopoulos et al. (41), Tjahyo et al. (86), Livingston et al. (87), Ni et al. (88), Zhang et al. (89), Agrinier et al. (90), and Rahimlou (91)

### 4.5 Precision nutrition: an emerging paradigm

An obvious difference exists in clinical outcomes after adopting different diets among individuals. Precision nutrition represents an emerging research direction aiming to incorporate individual-level information, such as genetic variants and gut microbial profiles, into dietary recommendations. However, its translation into routine clinical practice remains in early stages, and the evidence should be interpreted with appropriate caution. The carriers of PNPLA3 I148M and TM6SF2 E167K variants have an increased sensitivity to a high-fructose and refined-carbohydrate diet (92). Preliminary evidence suggests that these individuals may benefit from the aim to consume <25 g of fructose daily. They might consider selecting certain low-GI types of carbohydrate sources rather than high-GI varieties, although specific quantitative thresholds remain to be validated in prospective clinical trials.

From a practical standpoint, dietary management in MASLD/MAFLD can be conceptualized across three tiers of evidence maturity, reflecting the distinction between interventions that are currently actionable, those that may be cautiously considered based on phenotype, and those that remain investigational (93).

#### 4.5.1 Tier 1—currently actionable dietary quality improvement

This tier constitutes the evidence-based foundation of current practice and involves choosing the Mediterranean diet (MD) or the Dietary Approaches to Stop Hypertension (DASH) diet pattern (73, 94, 95), ensuring high-quality protein content (complementary to plant-based proteins) (35), balancing fatty acid ratios (n-6:n-3 < 4:1) and consuming >50% of whole grains (89). These recommendations are supported by existing dietary intervention studies and represent the current standard of care for MASLD/MAFLD (96).

#### 4.5.2 Tier 2—cautious phenotype-based adjustments

This tier acknowledges that persistent changes in hepatic steatosis can be introduced, e.g., time-restricted eating (TRE) with an 8-h eating window or a cycle diet of five meals per day minus one (80, 97, 98). Such approaches may be considered in selected patients based on their metabolic phenotype, disease severity, and individual treatment response, although their application should be guided by clinical judgment and monitoring rather than standardized protocols, as long-term comparative effectiveness data are still accumulating (93, 99).

#### 4.5.3 Tier 3—future precision strategies requiring validation

Research is actively exploring whether individualization based on genetic profiles (PNPLA3 and TM6SF2) and microbiome tests (16S ribosomal ribonucleic acid [rRNA] or metabolomics analysis) can improve clinical outcomes beyond conventional dietary counseling (100, 101). Preclinical and early-phase studies suggest potential benefits of strengthened fructose restriction and choline supplementation for carriers of these variants (100, 102) and the adoption of the combined probiotic and prebiotic intervention for dysbiosis (63, 103). Nevertheless, these strategies remain investigational. Genetic testing for PNPLA3 and TM6SF2 variants, 16S rRNA sequencing, metabolomics profiling, and targeted supplementation protocols have not yet been established as routine clinical pathways (104). Important unresolved questions include the clinical utility, cost-effectiveness, reproducibility across populations, and optimal implementation frameworks for such approaches. Their integration into standard dietary management for MASLD/MAFLD will require further validation through adequately powered, prospective randomized controlled trials (105, 106).

## 5 Discussion

### 5.1 Principal interpretation

This review conditionally interprets the “vegetarian hepatoprotection paradox”: low-quality plant-based diets (excessive in refined carbohydrates and fructose, deficient in high-quality protein, and imbalanced in the n-6:n-3 fatty acid ratio) may negate the anticipated hepatic benefits under specific metabolic and genetic contexts (102). This reframes the paradox as a matter of dietary quality rather than an indictment of vegetarianism per se.

The evidence does not support a monolithic hepatoprotective value of vegetarian diets. Six dietary configurations exhibit markedly divergent MASLD/MAFLD risk profiles (Table 1) (100, 102). The unhealthy plant-based dietary index (uPDI)—rich in refined grains, sweets, and ultra-processed vegetarian products—represents the highest-risk configuration despite complete animal product exclusion (102). Conversely, the healthy plant-based dietary index (hPDI) demonstrates inverse associations with metabolic syndrome and hepatic steatosis (22, 26, 28). It is dietary quality, not the categorical absence of animal products, that determines hepatic outcomes.

Four structurally deficient subpatterns have been identified: (i) high-refined-carbohydrate, low-protein patterns; (ii) n-6-dominant patterns (41, 42, 69); (iii) high-fructose, low-fiber patterns (46, 47, 107); and (iv) ultra-processed plant food–dependent patterns (108, 109). Each converges on hepatic lipid dysregulation through ChREBP-mediated DNL, impaired ApoB100-dependent VLDL assembly, and proinflammatory lipid mediator production (42, 69). However, these mechanisms derive predominantly from experimental and observational studies. Thus, direct causal attribution in human vegetarian populations remains inadequately tested (33, 110).

### 5.2 Why dietary quality matters more than vegetarian labeling

The critical insight emerging from this review is that dietary quality represents a more informative determinant of the MASLD/MAFLD risk than categorical vegetarian labeling (102). The binary “vegetarian” versus “non-vegetarian” classification obscures substantial within-group heterogeneity that may exceed between-group differences. Strict vegan and unhealthy plant-based diets occupy opposite ends of the hepatoprotective risk spectrum (100, 102), challenging the assumption that plant food exclusion alone confers a metabolic benefit.

The operative determinants of hepatic outcomes are the nutrient composition within a vegetarian pattern—refined versus whole carbohydrates, protein completeness, n-6:n-3 ratio, and fiber and polyphenol content (42, 69). This quality-centric perspective reconciles apparent contradictions in the literature: favorable outcomes are typically observed in whole-food plant-based populations (26, 30), whereas adverse effects are associated with refined carbohydrate–rich and processed vegetarian diets (21, 28). Future research should employ graded plant-based diet quality indices rather than binary classification (102).

### 5.3 Mechanistic plausibility and evidence boundaries

The mechanistic framework integrates three dimensions: upstream dietary factors, intermediate genetic susceptibility, and downstream gut microbiota configurations. Fructose-driven DNL and ChREBP-mediated lipogenesis achieve the highest certainty, supported by controlled feeding trial meta-analyses (107). However, directness to vegetarian populations remains indirect.

Gene-diet interactions involving PNPLA3 I148M and TM6SF2 E167K illustrate biological plausibility but low certainty. PNPLA3 I148M carriers may exhibit increased sensitivity to low-choline diets, which is particularly relevant to strict vegans (60, 61, 100). TM6SF2 E167K variants may interact with refined carbohydrates to impair VLDL-mediated triglyceride secretion (63, 64). The gut microbiota feedback loop (SCFA deficit, increased intestinal permeability, and LPS-TLR4-NF-κB activation) is biologically plausible (66, 103) but requires prospective validation in vegetarian cohorts.

### 5.4 Clinical translation and counseling implications

The evidence supports a shift from categorical labeling to quality-focused assessment (93, 104). Dietary management can be conceptualized across three evidence tiers. Tier 1 (actionable) includes Mediterranean or DASH patterns, high-quality protein complementation, n-6:n-3 < 4:1, and >50% of whole grains (72, 73, 76, 89). Tier 2 (phenotype-based) comprises time-restricted eating with an 8-h window (97, 98). Tier 3 (investigational) consists of genetic testing for PNPLA3/TM6SF2, microbiome profiling, and targeted supplementation (100, 101, 104, 111), which require randomized controlled trial validation (106) before clinical implementation.

Clinicians should distinguish fructose sources: adverse hepatic effects derive from sugar-sweetened beverages, fruit juices, and added sugars, but not from whole fruits containing fiber and polyphenols that attenuate fructose absorption (24, 107). Patients should minimize refined fructose while maintaining 2–3 daily servings of whole fruits.

### 5.5 Limitations and future research

As this is not a systematic review, purposive searches without formal risk-of-bias assessment render conclusions interpretive and hypothesis-generating (105, 110). The identified subpatterns are idealized constructs, and real-world diets rarely conform to single categories (93). Mechanistic links derive predominantly from animal models and non-vegetarian populations (100), and gene-diet-microbiome interactions remain incompletely validated (104). Precision nutrition strategies require prospective clinical confirmation.

Future research should prioritize graded plant-based diet quality indices in prospective cohorts; randomized controlled trials comparing high-quality plant-based diets with Mediterranean/DASH patterns (94); gene-diet interaction studies within vegetarian cohorts; prebiotic and probiotic interventions; and implementation science examining precision nutrition feasibility in clinical practice.

## 6 Conclusion

This critical narrative examines the conditions under which vegetarian dietary patterns may fail to confer expected hepatoprotection against MASLD/MAFLD. The central argument is that dietary quality—not categorical vegetarian labeling—determines hepatic metabolic outcomes. Four structurally deficient vegetarian subpatterns may converge on hepatic lipid dysregulation through mechanisms involving DNL, impaired VLDL assembly, and inflammatory pathway activation. These dietary factors may interact with genetic susceptibility variants, including PNPLA3 I148M and TM6SF2 E167K, and gut microbiota configurations to modulate the individual metabolic risk. However, this review does not establish that vegetarian diets cause MASLD/MAFLD; rather, it argues that low-quality vegetarian and plant-based patterns may, under certain metabolic and genetic conditions, fail to provide the hepatoprotection commonly assumed to accompany plant-food consumption.

The evidence supports a shift from categorical dietary labeling toward quality-focused assessment and intervention. Evidence-based dietary patterns, including the MD, the green MD, the DASH diet, and high-quality plant-based diets, offer structured approaches to MASLD/MAFLD management that can be adapted for vegetarian patients. Precision nutrition represents a promising but still investigational framework for individualized intervention. Future research should prioritize prospective studies using graded plant-based diet quality indices, randomized trials comparing dietary patterns in vegetarian cohorts, and gene-diet interaction studies to validate the mechanistic hypotheses advanced in this review.

## Glossary

AA: Arachidonic acid
AGA: American Gastroenterological Association
ALT: Alanine aminotransferase
AMPK: Adenosine monophosphate-activated protein kinase
AP-1: Activator protein-1
ApoB100: Apolipoprotein B-100
APRI: Aspartate aminotransferase-to-platelet ratio index
ATGL: Adipose triglyceride lipase
BA-MCY: Bile acid–methylcysteamine
BMI: Body mass index
ChREBP: Carbohydrate response element binding protein
CI: Confidence interval
DASH: Dietary Approaches to Stop Hypertension
DHA: Docosahexaenoic acid
DNL: *De novo* lipogenesis
EASL: European Association for the Study of the Liver
EASD: European Association for the Study of Diabetes
EASO: European Association for the Study of Obesity
EPA: Eicosapentaenoic acid
FXR: Farnesoid X receptor
GI: Glycemic index
GLUT5: Glucose transporter 5
hPDI: Healthy plant-based dietary index
HO-1: Heme oxygenase-1
IHF: Intrahepatic fat
IF: Intermittent fasting
IKKβ: IκB kinase
IL-6: Interleukin-6
JNK: c-Jun N-terminal kinase
KHK: Ketohexokinase
LPS: Lipopolysaccharide
LTB4: Leukotriene B4
MAFLD: Metabolic-associated fatty liver disease
MASLD: Metabolic dysfunction-associated steatotic liver disease
MD: Mediterranean diet
MLX: Max-like protein X
MUFAs: Monounsaturated fatty acids
NAFLD: Non-alcoholic fatty liver disease
NASH: Non-alcoholic steatohepatitis
NF-κB: Nuclear factor kappa-B
Nrf2: Nuclear factor erythroid-2-related factor 2
PFK-1: Phosphofructokinase-1
PGC-1*α*: Peroxisome proliferator-activated receptor gamma coactivator 1α
PGE2: Prostaglandin E2
PNPLA3: Patatin-like phospholipase domain-containing protein 3
PP2A: Protein phosphatase 2A
PPAR-α: Peroxisome proliferator-activated receptor-α
PUFAs: Polyunsaturated fatty acids
RCT: Randomized controlled trial
rRNA: Ribosomal ribonucleic acid
SCFAs: Short-chain fatty acids
SLC2A5: Solute carrier family 2 member 5
SREBP-1c: Sterol regulatory element-binding protein-1c
TG: Triglyceride
TMAO: Trimethylamine N-oxide
TLR4: Toll-like receptor 4
TNF-α: Tumor necrosis factor-alpha
TM6SF2: Transmembrane 6 superfamily member 2
TRE: Time-restricted eating
uPDI: Unhealthy plant-based dietary index
VLDL: Very low-density lipoprotein
VNN1: Vanin-1
WMD: Weighted mean difference
